# Neutralizing Antibody Responses following Long-Term Vaccination with HIV-1 Env gp140 in Guinea Pigs

**DOI:** 10.1128/JVI.00369-18

**Published:** 2018-06-13

**Authors:** Christine A. Bricault, James M. Kovacs, Alexander Badamchi-Zadeh, Krisha McKee, Jennifer L. Shields, Bronwyn M. Gunn, George H. Neubauer, Fadi Ghantous, Julia Jennings, Lindsey Gillis, James Perry, Joseph P. Nkolola, Galit Alter, Bing Chen, Kathryn E. Stephenson, Nicole Doria-Rose, John R. Mascola, Michael S. Seaman, Dan H. Barouch

**Affiliations:** a Center for Virology and Vaccine Research, Beth Israel Deaconess Medical Center, Boston, Massachusetts, USA; b Department of Chemistry and Biochemistry, University of Colorado, Colorado Springs, Colorado, USA; c Division of Molecular Medicine, Children's Hospital, Boston, Massachusetts, USA; d Department of Pediatrics, Harvard Medical School, Boston, Massachusetts, USA; e Vaccine Research Center, National Institute of Allergy and Infectious Diseases, National Institutes of Health, Bethesda, Maryland, USA; f Ragon Institute of MGH, MIT and Harvard, Boston, Massachusetts, USA; Emory University

**Keywords:** HIV-1, vaccine, neutralizing antibodies, long-term, multivalent, gp140, antibody function, human immunodeficiency virus, vaccines

## Abstract

A vaccination regimen capable of eliciting potent and broadly neutralizing antibodies (bNAbs) remains an unachieved goal of the HIV-1 vaccine field. Here, we report the immunogenicity of longitudinal prime/boost vaccination regimens with a panel of HIV-1 envelope (Env) gp140 protein immunogens over a period of 200 weeks in guinea pigs. We assessed vaccine regimens that included a monovalent clade C gp140 (C97ZA012 [C97]), a tetravalent regimen consisting of four clade C gp140s (C97ZA012, 459C, 405C, and 939C [4C]), and a tetravalent regimen consisting of clade A, B, C, and mosaic gp140s (92UG037, PVO.4, C97ZA012, and Mosaic 3.1, respectively [ABCM]). We found that the 4C and ABCM prime/boost regimens were capable of eliciting greater magnitude and breadth of binding antibody responses targeting variable loop 2 (V2) over time than the monovalent C97-only regimen. The longitudinal boosting regimen conducted over more than 2 years increased the magnitude of certain tier 1 NAb responses but did not increase the magnitude or breadth of heterologous tier 2 NAb responses. These data suggest that additional immunogen design strategies are needed to induce broad, high-titer tier 2 NAb responses.

**IMPORTANCE** The elicitation of potent, broadly neutralizing antibodies (bNAbs) remains an elusive goal for the HIV-1 vaccine field. In this study, we explored the use of a long-term vaccination regimen with different immunogens to determine if we could elicit bNAbs in guinea pigs. We found that longitudinal boosting over more than 2 years increased tier 1 NAb responses but did not increase the magnitude and breadth of tier 2 NAb responses. These data suggest that additional immunogen designs and vaccination strategies will be necessary to induce broad tier 2 NAb responses.

## INTRODUCTION

Successful elicitation of broadly neutralizing antibodies (bNAbs) against the HIV-1 envelope (Env) protein through vaccination remains an important but unachieved goal. It is known that 15 to 20% of individuals chronically infected with HIV-1 are capable of eliciting bNAbs (1–4). These individuals first develop NAbs (5, 6), which drive viral escape and evolution, resulting in expansion of Env diversity (4, 7–9). In some patients, this sequence diversity drives the development of bNAbs capable of targeting conserved epitopes (10–13). These studies suggest that the long-term exposure of the immune system to multiple diverse Env sequences can result in the development of bNAbs.

No HIV-1 vaccine to date has been capable of eliciting bNAbs in humans (14–17). A variety of strategies have been explored with the goal of expanding the breadth of vaccine-elicited NAbs. One strategy assessed mixtures of different Envs with the goal of exposing B cells to sequence diversity, but this approach did not appreciably improve the breadth of tier 2 NAb responses (18–23). Additionally, groups have utilized rationally designed immunogens focused on eliciting bNAbs to a single Env epitope, such as the CD4 binding site, but these immunogens have not driven the full development of such bNAbs (24–26). Mimics of the native HIV-1 Env trimer, such as the SOSIP trimer, have also been assessed, but they elicited NAbs with minimal breadth and targeted a hole in the glycan shield (27–30). Finally, long-term vaccination strategies have been considered, with the goal of allowing for affinity maturation and the development of neutralization breadth. A few studies have explored vaccination regimens spanning multiple years; however, they have also failed to induce broad tier 2 neutralization (31).

In this study, we evaluated the effects of a longitudinal prime/boost vaccination regimen on the evolution of binding and NAb responses in guinea pigs over a vaccination regimen that spanned more than 2 years. We found that multivalent, sequential prime/boost vaccination regimens improved the breadth of binding antibodies compared to vaccination with a single Env. Additionally, while we observed a limited breadth of tier 2 NAbs in all vaccination regimens, the breadth and magnitude of these NAbs did not increase over the course of the longitudinal regimen. These data suggest that novel immunogen design strategies and vaccination regimens will be needed to improve tier 2 NAb responses.

## RESULTS

### Longitudinal vaccination regimens.

**FIG 1:**
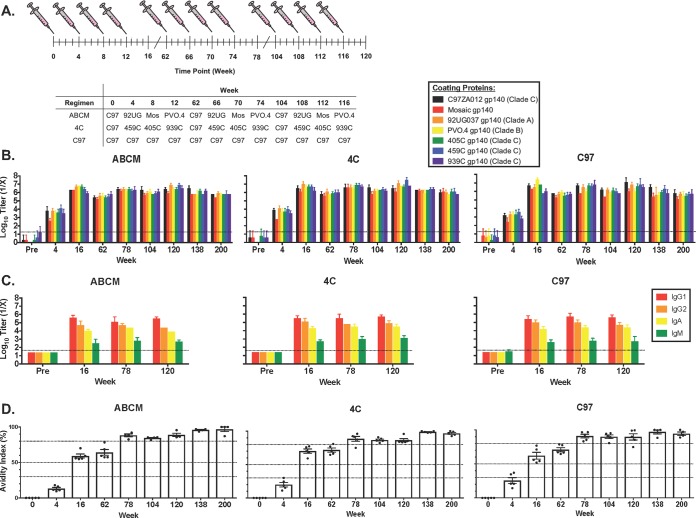
Vaccination regimens and characterization of binding antibody responses. (A) Vaccination regimens for guinea pigs immunized with a longitudinal prime/boost vaccination schedule. Animals were vaccinated at weeks 0, 4, 8, 12, 62, 66, 70, 74, 104, 108, 112, and 116 utilizing the listed vaccination regimens and bled 4 weeks after each vaccination, as well as at weeks 138 and 200. C97, C97ZA012 gp140; 92UG, 92UG037 gp140; Mos, mosaic gp140. Error bars represent the standard deviations. (B) Binding antibody titers for HIV-1 Env gp140s of different clades as measured by endpoint ELISAs. (C) Binding antibody titers for HIV-1 C97ZA012 gp140, showing specific isotype and subclass responses, as measured utilizing endpoint ELISAs. Error bars represent the standard deviations. (D) Guinea pig polyclonal antibody avidity as measured by urea disruption ELISA. Each dot represents the result for an individual animal, and error bars represent the standard deviations. Percent avidity was calculated using the following formula: [(absorbance of urea-treated sample/absorbance of non-urea-treated matched sample) × 100]. Zero to 30% is low avidity, 30 to 50% is moderate avidity, and >50% is high avidity. The 80% bar is used as a reference point within the high-avidity region.

Our laboratory has previously generated HIV-1 Env gp140 immunogens from clades A (92UG037), B (PVO.4), and C (C97ZA012, 405C, 459C, and 939C), as well as a bioinformatically optimized mosaic immunogen (Mosaic 3.1) (20, 21, 32–34). These gp140s elicited robust binding and tier 1 NAb responses in small animal models. We evaluated whether repeated boosting with homologous or heterologous gp140 Env immunogens over a prolonged period of time would increase the magnitude and breadth of NAb responses. Three vaccine regimens were evaluated: C97ZA012 gp140 alone (C97; *n* = 5), clade A, B, C, and mosaic gp140s (92UG037, PVO.4, C97ZA012, and Mosaic 3.1, respectively [ABCM]; *n* = 5), and four clade C gp140s (C97ZA012, 459C, 405C, and 939C [4C]; *n* = 5) (Fig. 1A). Vaccines were given sequentially as single Env immunogens at weeks 0, 4, 8, 12, 62, 66, 70, 74, 104, 108, 112, and 116, and peak immunogenicity was assessed at weeks 16, 78, and 120.

### Binding antibody responses.

We first assessed the ability of each vaccination regimen to elicit binding antibodies to a multiclade panel of vaccine-matched gp140 proteins by enzyme-linked immunosorbent assay (ELISA) (Fig. 1B). All guinea pigs elicited comparable and robust binding antibody responses after the second vaccination. The highest titers were seen at weeks 16, 78, and 120, which correspond to peak immunogenicity, and there were contractions in the overall Env-specific binding antibody responses after long-term rests (weeks 62, 104, 138, and 200). For all groups, the IgG1 responses were the highest, followed by the IgG2, IgA, and IgM responses (Fig. 1C). Antibody avidity in all groups increased similarly over time, peaking at week 78, where it plateaued until week 200 (Fig. 1D). Mucosal IgG responses were also detected in the majority of animals, but at lower titers (data not shown).

### Mapping binding antibody responses.

**FIG 2:**
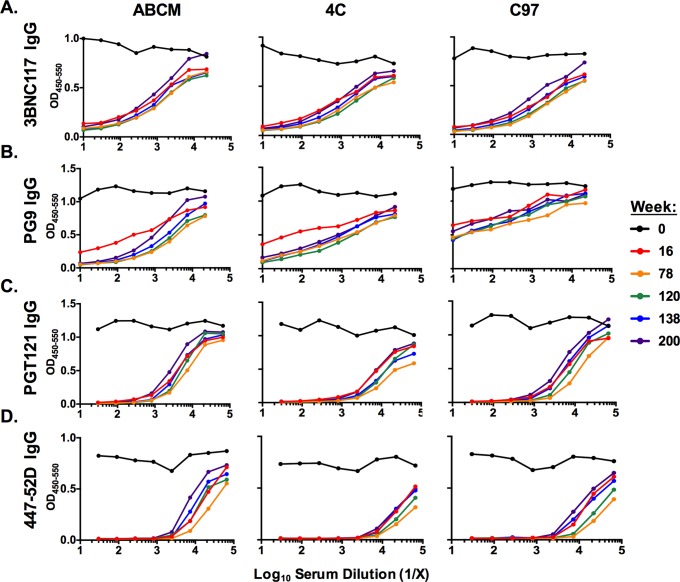
Mapping of polyclonal binding antibody responses by competition ELISA. Competition ELISAs showing the ability of polyclonal guinea pig sera to outcompete binding of 3BNC117 IgG (A), PG9 IgG (B), PGT121 IgG (C), and 447-52D IgG (D) human monoclonal antibodies to HIV-1 Env gp140 coating antigens. Guinea pig vaccination regimes, as shown in Fig. 1A, are indicated at the top. Data for different vaccination time points are shown as indicated in the key.

We mapped binding antibody responses by competition ELISAs using the following bNAbs: 3BNC117 to the CD4 binding site (CD4bs) (35), PG9 to variable loop 2 (V2)/glycans (36), PGT121 to V3/glycans (37), and 447-52D to V3 (38). Sera from all groups outcompeted 3BNC117 binding to C97 gp140 similarly, suggesting that all vaccine regimens elicited binding antibodies in the vicinity of the CD4bs (Fig. 2A). The ABCM and 4C regimens were able to outcompete PG9 binding more successfully than the C97 regimen, with this effect increasing from week 16 to 78 (Fig. 2B). The 4C regimen was superior to the C97 regimen at outcompeting PGT121 and 447-52D binding (Fig. 2C and D). These data suggest that while the binding antibodies target multiple regions of Env, the 4C regimen induced the most robust V3-directed binding antibodies.

### Assessing the magnitude of binding antibodies to linear peptides across the HIV-1 envelope.

**FIG 3:**
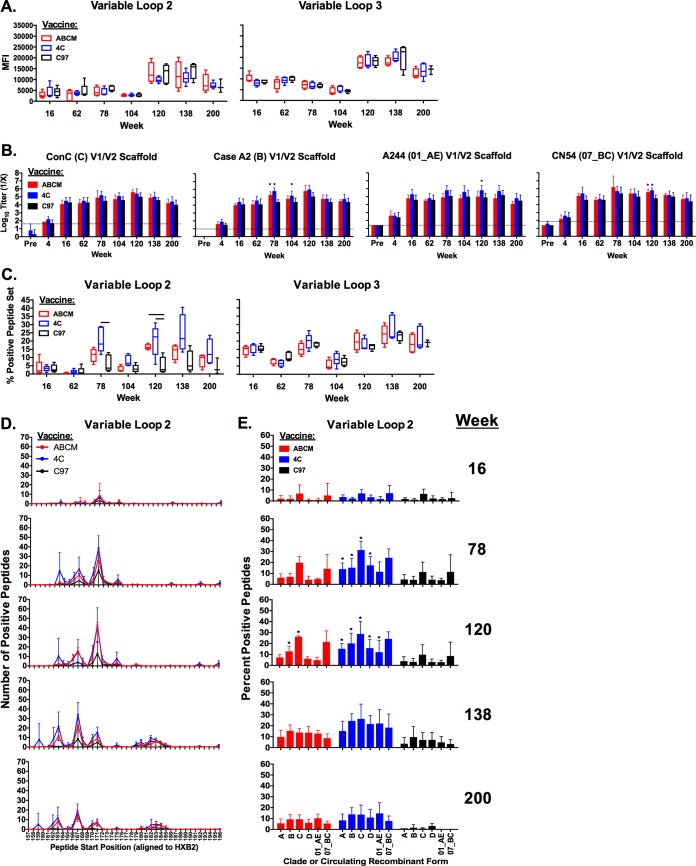
Binding antibodies to linear peptides elicited in longitudinally vaccinated guinea pigs. (A and B) Magnitudes of antibody responses in sera from vaccinated guinea pigs to linear peptides in variable loops 1, 2, and 3. (A) Magnitudes of antibody binding to peptide microarray as defined by mean fluorescence intensities (MFI) of signals. Envelope regions targeted are shown at the top. (B) Binding antibody titers to variable loop 1 and 2 scaffolds as determined by endpoint ELISAs. Dotted line indicates background signal, and error bars indicate standard deviations. Pre, naive sera. Asterisks indicate results for ABCM- or 4C-vaccinated animals that were statistically significantly different from the results for C97-vaccinated animals at the matched time points (*P* < 0.05, Mann-Whitney U test). (C) Percentages of positive peptides as measured by peptide microarray. Percentage of positive peptides is defined as follows: [(positive peptides within a region/total number of peptides within a region) × 100]. Black bars indicate results for ABCM- or 4C-vaccinated guinea pigs that were statistically significantly different from the results for C97-vaccinated animals at the matched time points (*P* < 0.05, Mann-Whitney U test). (D) Binding distribution of binding antibodies across V2 peptides as determined by peptide microarray. (E) Binding distribution of binding antibodies across select clades and circulating recombinant forms (CRF) of HIV-1 Env V2 peptides as determined by microarray. Error bars show standard deviations. Asterisks indicate results for ABCM- or 4C-vaccinated guinea pigs that were statistically significantly different from the results for C97-vaccinated animals at a matched time point and clade/CRF (*P* < 0.05, Mann-Whitney U test).

We next mapped linear epitope binding antibodies to HIV-1 gp140 elicited by each vaccination regimen by utilizing a peptide microarray (39). The majority of early responses were elicited to V3 at week 16, although responses to V2 were boosted over the course of the vaccination regimen and persisted to week 200 (Fig. 3A). Interestingly, there were large increases in the magnitude of linear binding responses for both V2 and V3 from week 104 to week 120 that gradually declined until week 200. We also found that all vaccination regimens also elicited robust V1/V2 binding antibodies using V1/V2 scaffolds (Fig. 3B) (40, 41). Furthermore, at weeks 78 and 120, the 4C regimen elicited binding antibodies to a statistically greater number of V2 peptides than C97 alone (Mann-Whitney U test, *P* < 0.05). Similarly, the ABCM vaccine elicited binding antibodies to a statistically superior number of V2 peptides at week 120 than C97 alone (Mann-Whitney U test, *P* < 0.05) (Fig. 3C). All vaccines elicited binding antibodies to similar regions of V2 across all time points, with the multivalent vaccines eliciting antibodies to greater numbers of peptides within these regions at weeks 78, 120, and 138 (Fig. 3D). Additionally, the multivalent vaccines elicited binding antibodies capable of binding to greater numbers of sequences within different clades and circulating recombinant forms (CRFs) than did C97 vaccines (Mann-Whitney U test, *P* < 0.05) (Fig. 3E).

### Tier 1 neutralizing antibody responses.

**FIG 4:**
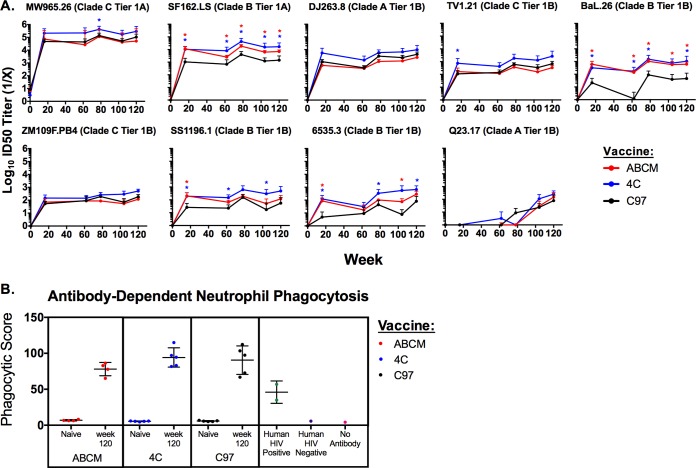
Magnitudes of functional neutralizing and functional non-neutralizing antibody responses. (A) Magnitudes of heterologous tier 1 NAb titers elicited in vaccinated guinea pigs. Guinea pig sera obtained prevaccination (week 0) and at weeks 16, 62, 78, 104, and 120 were tested against a multi-clade panel of tier 1 isolates in the TZM.bl neutralization assay. Groups were vaccinated with the ABCM (*n* = 4), 4C (*n* = 5), or C97 (*n* = 5) regimen. The test pseudovirus, its tier, and clade are shown above each panel. The MuLV background signal for each animal-matched MuLV control was subtracted from neutralization data for all data points. Error bars show the standard deviation for each group at each time point. The limit of detection for the assay is a 50% infective dose (ID_50_) titer of 20. Asterisks denote statistically significant differences from the results for C97 vaccination alone (*P* < 0.05, Mann-Whitney U test). (B) Magnitudes of antibody-dependent neutrophil phagocytosis (ADNP) for guinea pig samples. Guinea pig sera obtained prevaccination (naive) and at week 120 were tested in an ADNP assay as described in Materials and Methods. Groups were vaccinated with the ABCM (*n* = 4), 4C (*n* = 5), or C97 (*n* = 5) regimen. Each dot represents a sample tested in triplicate, and error bars show standard deviations for each vaccination group. Controls included human HIV-positive serum, human HIV-negative serum, and no-antibody samples. Phagocytic score was determined using the following formula: (% bead-positive SSC^high^, CD11R1^+^, and CD4^−^ cells × MFI of bead-positive cells)/10,000.

We next assessed the neutralization capacity of the antibodies utilizing a multiclade tier 1 panel of neutralization-sensitive pseudoviruses using the TZM.bl neutralization assay (42, 43). Against specific tier 1 pseudoviruses (SF162.LS, BaL.26, SS1196.1, and 6535.3), the 4C and ABCM regimens generally elicited higher NAb titers than did the C97 regimen (Mann-Whitney U test, *P* < 0.05) (Fig. 4A). For one pseudovirus, Q23.17, no neutralization was observed at week 16 but clear neutralization emerged by week 120, indicating that repetitive boosts over a prolonged period of time can increase the breadth of tier 1 NAbs. However, the NAb titers against most tier 1 pseudoviruses assessed did not increase over time.

### ADNP responses.

We next assessed antibody-dependent neutrophil phagocytosis (ADNP) responses as a measure of functional, nonneutralizing antibody responses. All vaccination groups elicited high ADNP responses at week 120 (Fig. 4B). No differences in the magnitudes of ADNP responses were observed among the different vaccines tested.

### Tier 2 neutralizing antibody responses.

**FIG 5:**
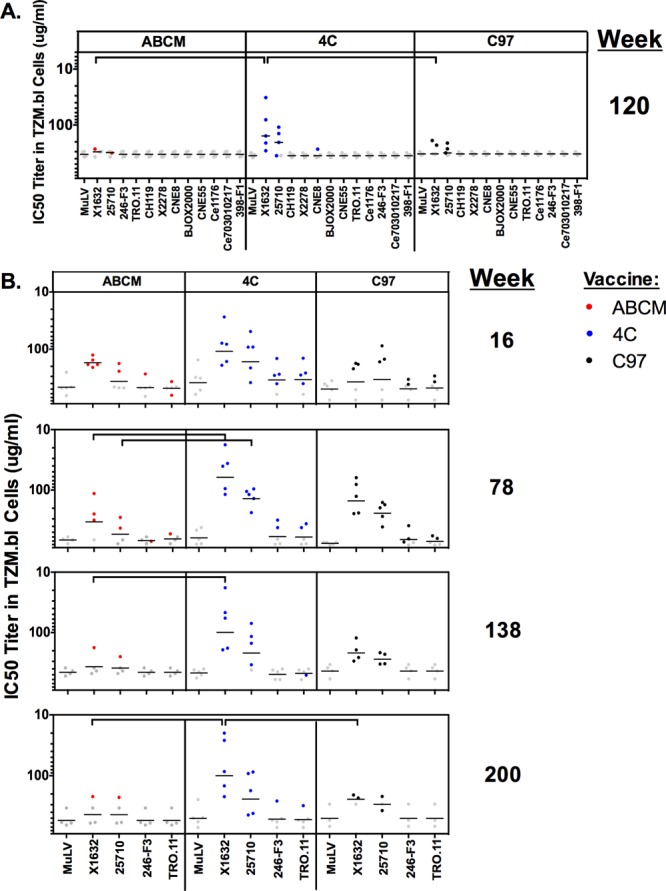
Magnitudes of heterologous tier 2 NAb activities in purified IgGs elicited in vaccinated guinea pigs. Purified polyclonal IgGs from vaccinated guinea pigs were evaluated against select tier 2 pseudoviruses and MuLV (negative control) at week 120 against the full tier 2 global panel (A) and at weeks 16, 78, 138, and 200 against select tier 2 viruses in the global panel (B). Horizontal black lines indicate mean titers, and gray dots indicate responses below the limit of detection of the assay. Red, blue, and black dots represent responses that were both detectable and greater than the value for the MuLV control. Each dot represents the result for a single guinea pig, colored according to vaccination regimen. In the C97 vaccination group, one guinea pig died at week 134 and one at week 177 due to age. Horizontal brackets denote statistically significantly different results (*P* < 0.05, Student's *t* test).

We next assessed NAb responses to tier 2 viruses, which are representative of circulating strains of HIV-1 and are more neutralization resistant than tier 1 viruses. For these studies, we utilized a defined, global, tier 2 panel of pseudoviruses selected to represent global HIV-1 sequence diversity (44). Purified IgG was used to reduce the low nonspecific background signal in the assay. We observed positive NAb responses to tier 2 pseudoviruses X1632 (clade G) and 25710 (clade C) for the week 120 samples, with the highest-magnitude responses in the 4C group (Student's *t* test, *P* < 0.05 for X1632) (Fig. 5A). We evaluated the kinetics of the modest tier 2 responses over time and found that these responses were initially raised at week 16 for all vaccines tested (Fig. 5B). By week 78, the 4C regimen elicited higher NAb responses than the ABCM group against X1632, and this result was maintained for the duration of the entire study (Student's *t* test, *P* < 0.05). The 4C regimen also elicited higher NAb responses than the ABCM group against 25710 at week 78. Additionally, the 4C group elicited higher NAb titers than the C97 group against X1632 at weeks 120 and 200 (Student's *t* test, *P* < 0.05) (Fig. 5B). The tier 2 responses did not increase in magnitude or breadth over the course of the vaccination regimen. Thus, all vaccination regimens elicited limited and modest tier 2 NAbs, particularly the 4C regimen, but repetitive boosting over 2 years was unable to expand these tier 2 NAbs.

### Mapping the specificities of tier 2 neutralizing antibody responses.

**FIG 6:**
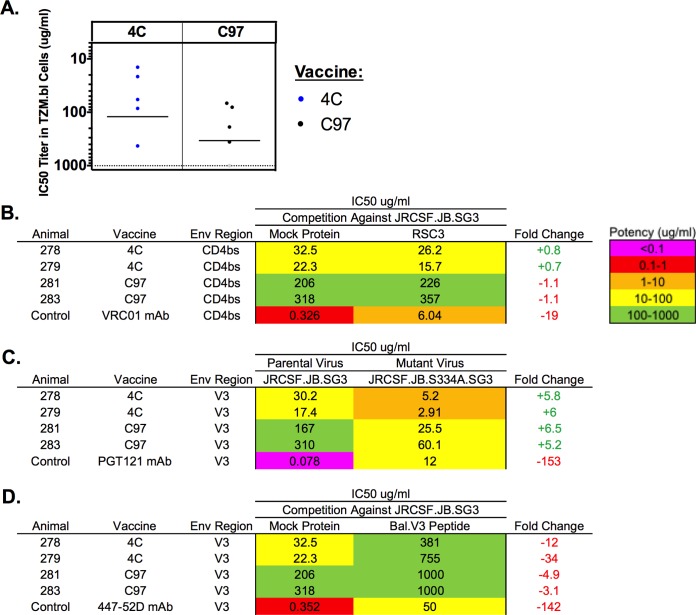
Mapping of tier 2 neutralizing antibody responses. (A) Purified polyclonal IgGs from vaccinated guinea pigs were evaluated against the tier 2 pseudovirus JRCSF. Horizontal black lines indicate mean titers, gray dots indicate responses below the limit of detection of the assay, and blue and black dots represent responses that were both detectable and greater than the limit of detection. Each dot represents the result for a single guinea pig. (B to D) Mapping was conducted with CD4 binding site competition by RSC3 (B), on a V3 glycan knockout virus (S334A) (C), and with V3 peptide competition (D). Tables show neutralization potencies against wild-type virus compared to mutant virus or against a virus with and without competition. +, increased neutralization potency; −, decreased neutralization potency.

Finally, we conducted functional mapping studies to determine the targets of the tier 2 NAbs. Both the 4C and C97 regimens elicited detectable neutralization titers to tier 2 virus JRCSF (Fig. 6A). Animals that elicited the highest NAb responses to JRCSF were utilized for mapping studies (guinea pigs 278, 279, 281, and 283). This tier 2 neutralization could not be outcompeted by RSC3 (CD4bs target) (Fig. 6B) or by using JRCSF with an S334A mutation (encoding a change from S to A at position 334; V3 target) (Fig. 6C), suggesting minimal CD4bs or V3/glycan-directed neutralization. In contrast, the neutralization of JRCSF was largely but not completely competed by a V3 linear peptide from Bal (Fig. 6D).

## DISCUSSION

In this study, we evaluated the immunogenicity of repeated boosting with homologous and heterologous Env gp140 vaccination regimens over the course of more than 2 years in guinea pigs. The multivalent 4C and ABCM regimens elicited greater breadths of V2-binding antibodies than the monovalent C97 regimen. Limited and modest heterologous tier 2 NAbs were also induced, particularly by the 4C regimen, with responses largely directed against a linear epitope in V3. Tier 2 NAbs did not increase in magnitude or breadth following repeated boosting over the 120-week vaccination regimen. These data suggest that novel immunogen design and vaccination strategies will likely be needed to induce broad, high-titer tier 2 NAbs.

Few HIV-1 vaccination studies have explored vaccination regimens spanning more than 2 years in duration. Saunders et al. conducted a study over a 4-year period which was capable of eliciting glycan-dependent, V3-directed NAbs after repeated vaccination with a single Env sequence in rhesus monkeys (31). These monkeys elicited tier 2 NAbs to kifunensine-treated pseudoviruses, but not to viruses containing wild-type glycosylation patterns. Our study, in contrast, utilized both homologous and heterologous prime/boost regimens in guinea pigs to assess the role that multivalency may play in improving NAb responses, and the 4C vaccine improved the achieved neutralization magnitude over the course of a long-term vaccination regimen. Animals elicited V3-directed NAbs able to neutralize wild-type tier 2 pseudoviruses, and neutralization was not dependent on the S334 glycan. Our study, however, was limited, as four Env sequences do not represent the total sequence diversity of viruses that would be found in an HIV-1-infected individual capable of eliciting bNAbs, and increased sequence diversity may be required to drive bNAb breadth (45, 46).

Interestingly, over the course of the longitudinal vaccination regimen, antibodies to linear epitopes and tier 1 NAbs evolved, while the tier 2 NAb responses remained largely unchanged over time. While there were increases in the breadth of V2-binding antibodies and tier 1 NAbs, this did not result in an increased magnitude or breadth of tier 2 NAbs. These data suggest that a longitudinal vaccination alone will not serve to drive tier 2 neutralization breadth. The limited tier 2 NAb responses induced in this study were largely directed to a linear epitope within the V3 loop. This is not surprising, given that V3 is an immunodominant epitope within the Env protein (47) and that gp140 immunogens sample various conformations, some of which expose V3 (48, 49). Monoclonal antibodies that target linear epitopes in V3 against a limited breadth of tier 2 viruses have previously been characterized, including 447-52D and 3074 (38, 50). Similar V3-directed NAbs have also been isolated from HIV-1-infected individuals and have been elicited in a number of vaccine studies (27, 28, 51–53). Utilizing strategies to reduce V3 exposure and immunogenicity may assist in increasing the breadth of tier 2 NAb responses. Studies with native-like SOSIP trimers, however, have to date not been able to expand the breadth of tier 2 NAb responses, and thus, utilizing a native-like trimer alone is not sufficient to increase the breadth of NAb responses.

Recently, it has been shown that immunization of cows readily generates broad NAbs, likely as a result of their unique germ line antibody repertoires (54). We believe that guinea pigs are a better model for predicting immunogencity in primates and humans, as studies with both foldon and SOSIP Env gp140s have shown comparable antibody profiles in guinea pigs and primates (32, 55).

Future immunogen design strategies will likely need to include modifications to limit V3 exposure, as well as to prime neutralizing responses to a greater number of epitopes. A variety of strategies exist to minimize V3 exposure, including using a SOSIP Env immunogen (27, 28, 56). These immunogens, however, elicit minimal heterologous tier 2 NAbs despite being accurate mimics of the HIV-1 Env trimer. Therefore, rational immunogen designs paired with SOSIP trimers may be beneficial for improving tier 2 NAb responses to pair stabilizing mutations with improved sequence designs. Concepts to improve neutralization breadth, such as utilizing immunogens that target the germ line precursors, assessing strategies to increase somatic hypermutation, and using bioinformatics approaches to create multivalent immunogen cocktails, paired with longitudinal boosting, should continue to be explored.

## MATERIALS AND METHODS

### Plasmids, cell lines, protein production, and antibodies.

The codon-optimized synthetic genes for all HIV-1 Env gp140s were produced by GeneArt (Life Technologies). All constructs contained a consensus leader signal sequence peptide, as well as a C-terminal foldon trimerization tag followed by a His tag (32, 57).

All gp140 proteins were expressed in 293T cells utilizing stable cell lines (for C97ZA012, 92UG037, and Mosaic 3.1) (Codex Biosolutions) or transient transfections with polyethylenimine (for 405C, 459C, 939C, and PVO.4) (20, 21, 33). His-tagged proteins were purified by using a HisTrap Ni-nitrilotriacetic acid (NTA) column (GE Healthcare). Ni-NTA columns were washed with 20 mM imidazole (pH 8.0), and protein was eluted with 300 mM imidazole (pH 8.0). Fractions containing protein were pooled and concentrated. Protein constructs were further purified utilizing gel filtration chromatography on Superose 6 (GE Healthcare) in running buffer containing 25 mM Tris (pH 7.5) and 150 mM sodium chloride. Purified proteins were concentrated using CentriPrep YM-50 concentrators (Millipore), flash frozen in liquid nitrogen, and stored at −80°C.

NAb 3BNC117 was provided by Michel Nussenzweig (Rockefeller University, New York, NY). NAbs PG9 and 447-52D were purchased from Polymun Scientific. NAb PGT121 was purchased from Catalent. Gp70 V1/V2 HIV-1 envelope scaffolds, including ConC, Case A2, CN54, and A244 V1/V2, were purchased from Immune Technology Corp.

### Guinea pig vaccinations.

Outbred female Hartley guinea pigs (Elm Hill) were used for all vaccination studies and were housed at the Animal Research Facility of Beth Israel Deaconess Medical Center under approved Institutional Animal Care and Use Committee (IACUC) protocols.

Guinea pigs (*n* = 5/group) were immunized with Env protein intramuscularly in the quadriceps bilaterally at 4-week intervals, with two long-term rests, for a total of 12 injections. Vaccination groups included C97ZA012 gp140 only (C97, *n* = 5); a sequential prime/boost regimen, including clade C C97ZA012, clade A 92UG037, Mosaic 3.1, and clade B PVO.4 gp140s (ABCM, *n* = 5); and clade C C97ZA012, 459C, 405C, and 939C gp140s (4C, *n* = 5) (Fig. 1A). For ABCM, one animal died following blood draw and was excluded from the analysis. One guinea pig died at week 134 and one at week 177 in the C97 vaccination group due to age.

Twelve vaccinations were given as three sets of 4 immunizations at weeks 0, 4, 8, and 12, at weeks 62, 66, 70, and 74, and at weeks 104, 108, 112, and 116 (Fig. 1A). Animals were followed for a total of 200 weeks. Vaccinations consisted of a total of 100 μg of Env gp140 per injection formulated in 15% Emulsigen (vol/vol) oil-in-water emulsion (MVP Laboratories) and 50 μg CpG (Midland Reagent Company) as adjuvants. Serum samples were obtained from the vena cava of anesthetized animals.

### Endpoint ELISAs.

Serum binding antibodies against gp140 and V1/V2 scaffolds were measured by endpoint enzyme-linked immunosorbent assays (ELISAs) (32). Briefly, ELISA plates (Thermo Scientific) were coated with individual gp140s or V1/V2 scaffolds and incubated overnight. For isotype and subclass ELISAs, plates were coated with C97ZA012 gp140. Guinea pig sera were then added in serial dilutions and detected with a horseradish peroxidase (HRP)-conjugated goat anti-guinea pig secondary antibody for total IgG ELISAs (Jackson ImmunoResearch Laboratories). For isotyping ELISAs, HRP-conjugated goat anti-guinea pig IgG1, goat anti-guinea pig IgG2, goat anti-guinea pig IgM, and sheep anti-guinea pig IgA were utilized (MyBiosource). Plates were developed and read using the SpectraMax plus ELISA plate reader (Molecular Devices) and SoftMax Pro 4.7.1 software. Endpoint titers were considered positive at the highest dilution that maintained an absorbance that was >2-fold above background values.

### Avidity ELISAs.

Serum binding antibody avidities to HIV-1 Env gp140 were measured by a urea disruption enzyme-linked immunosorbent assay (ELISA) (58). Briefly, ELISA plates (Thermo Scientific) were coated with individual Env gp140s and incubated overnight. Guinea pig sera were prepared in a dilution plate to values between 1.0 and 1.5 at optical densities of 450 nm (OD_450_) to OD_800_ to provide antibody concentrations within a linear range. Sera were plated in duplicate twice; one duplicate was treated with 8 molar urea (Sigma-Aldrich) and the other with distilled water as a control. Plates were then incubated with an HRP-conjugated goat anti-guinea pig secondary antibody (Jackson ImmunoResearch Laboratories). Plates were developed and read using the SpectraMax plus ELISA plate reader (Molecular Devices) and SoftMax Pro 4.7.1 software (OD_450_ to OD_800_). Percent avidity was calculated using the following formula: [(average absorbance of urea-treated sample/average absorbance of water-treated, matched sample) × 100]. The avidity index describes 0 to 30% as low-avidity, 30 to 50% as moderate-avidity, and >50% as high-avidity binding antibodies.

### Competition ELISAs.

NAbs PG9, 3BNC117, PGT121, and 447-52D IgG were biotinylated using the EZ-Link micro NHS-PEG_4_ (*N*-hydroxysuccinimide ester–4-unit polyethylene glycol) biotinylation kit (Thermo Scientific) following the manufacturer's instructions. Antibodies were used at readings of approximately 1.0 to 1.5 at OD_450_ to OD_550_ for each coating protein. For PG9 IgG competition, Mosaic 3.1 Env gp140 was utilized as the coating protein, and for 3BNC117, PGT121, and 447-52D IgG competition, C97ZA012 Env gp140 was utilized as the coating protein. ELISA plates (Thermo Scientific) were coated overnight with gp140s. Guinea pig sera in blocking buffer (1% bovine serum albumin, 1× phosphate-buffered saline, 0.05% Tween) were added at a 1:10 dilution and serially diluted at 1:3 dilutions down the plate. Week 0, 16, 78, 120, 138, and 200 samples were run side-by-side on the same plate. A single biotinylated monoclonal IgG was then added at a single concentration. Streptavidin-HRP (Thermo Scientific) in blocking buffer was then added. Plates were then developed with SureBlue tetramethylbenzidine (TMB) microwell peroxidase substrate and TMB stop solution (Kirkegaard & Perry Laboratories, Inc.). Plates were developed and read at OD_450_ to OD_550_ using the SpectraMax plus ELISA plate reader (Molecular Devices) and SoftMax Pro 4.7.1 software.

### Peptide microarrays.

Peptide arrays were generated, assays conducted, and data analyzed using described previously methods (39). RepliTope antigen collection HIV ultra slides (JPT Peptide Technologies GmbH) were utilized. Each slide contains 6,654 15-mer peptides, printed in triplicate (subarrays), overlapping by 11 amino acids, representing 135 different clades or circulating recombinant forms (CRFs) that cover the entire HIV-1 genome. Microarray slides were incubated with guinea pig sera diluted 1/200 in SuperBlock T20 (Tris-buffered saline [TBS]) blocking buffer (Thermo Scientific). Alexa Fluor 647-conjugated AffiniPure goat anti-guinea pig IgG(H+L) was used as the secondary detection antibody (Jackson ImmunoResearch Laboratories). All batches of slides with the same time point were run in parallel with a control slide incubated with the secondary antibody only.

Slides were scanned with a GenePix 4300A scanner (Molecular Devices), using 635-nm and 532-nm lasers. The fluorescence intensity for each feature (peptide spot) and threshold values for positivity were calculated using GenePix Pro 7 software and GenePix Array List as described previously (39, 59). The fluorescence signal from a control slide incubated with just the secondary fluorophore was subtracted from the fluorescence signals from all experimental slides to remove the background signal associated with the fluorophore. For each batch of slides run together, the highest *P* value from all arrays run that was <10^−16^ was chosen as the cutoff for all slides for stringency (21, 60). All *P* values that fell below the cutoff *P* value of <10^−16^ were set to equal zero, and all samples with *P* values greater than the cutoff value were maintained as their raw, positive signal. Statistically significant differences between the results for guinea pigs that received the C97 prime/boost regimen and for animals that received either the ABCM or 4C prime/boost regimen at a matched time point (*P* < 0.05, Mann-Whitney U test) were determined using Prism 7.0 (GraphPad).

### TZM.bl neutralization assay.

Functional neutralizing antibody responses against HIV-1 Env pseudoviruses were measured using a luciferase-based virus neutralization assay in TZM.bl cells as described previously (42). Murine leukemia virus (MuLV) was included in all assays as a negative control. For graphing tier 1 pseudovirus data, responses were considered positive if they were greater than the MuLV value and were greater than 0 after MuLV background subtraction. Pseudoviruses were prepared as described previously (42, 61). Statistical analyses were conducted on tier 1 pseudovirus neutralization responses utilizing Prism 7.0 (*P* < 0.05, Mann-Whitney U test) (GraphPad).

For assessing neutralization against tier 2 pseudoviruses, polyclonal IgGs were purified from sera to reduce the background signal. High-capacity protein A agarose (Thermo Scientific) was used, following the manufacturer's instructions, and buffer exchanged into 1× phosphate-buffered saline (PBS), pH 7.4 (Gibco). Purified polyclonal IgGs were used in the TZM.bl assay against the global tier 2 panel (44) at week 120, which was the terminal peak time point. As animals only showed positivity against X1632 and 25710, purified polyclonal IgGs from the remaining peak time points were run against select tier 2 pseudoviruses. Statistical analyses of tier 2 neutralization responses were conducted utilizing Prism 7.0 (*P* < 0.05, Student's *t* test) (GraphPad).

For V3 mapping, a glycan mutant of JRCSF.JB containing an S334A glycan knockout mutation (JRCSF.JB.S334A) and JRCSF.JB with Bal.01 V3 for peptide competition were utilized. For CD4 binding site (CD4bs) mapping, JRCSF.JB with RSC3 competition was used (62).

### Antibody-dependent guinea pig neutrophil phagocytosis.

C97ZA012 gp140 was biotinylated and coupled to red fluorescent beads (Thermo Scientific). Sera from vaccinated or naive guinea pigs were diluted 1:12,500 in Roswell Park Memorial Institute (RPMI) 1640 medium and 10% fetal bovine serum (FBS) culture medium and incubated with gp140-coupled beads for 2 h at 37°C. Guinea pig white blood cells were isolated from EDTA-treated whole blood from naive guinea pigs. Red blood cells were lysed in ammonium chloride-potassium (ACK) lysis buffer, and white blood cells were collected by centrifugation, washed 3 times with 1× PBS, and resuspended to a volume of 5 × 10^5^ cells/ml in RPMI 1640–10% FBS culture medium. Diluted serum and bead complexes were pelleted at 1,000 rotations per minute for 10 min, the supernatant removed, and 5 × 10^4^ cells/well of guinea pig white blood cells were added and incubated for 1 h at 37°C. Following incubation, cells were washed with 1× PBS and stained with blue viability dye for 15 min at 4°C. Cells were stained with anti-CD11R1 antibody (clone MIL4; Bio-Rad) and anti-CD4 antibody (clone CT7; Bio-Rad) for 15 min at room temperature. Cells were washed twice with 1× PBS, fixed with 4% paraformaldehyde, and analyzed for phagocytosis by flow cytometry (BD LSR Fortessa and FACSDiva). Neutrophils were defined as side scatter high (SSC^high^), CD11R1^+^, and CD4^−^ as described previously (63), using FlowJo analysis software, and a phagocytic score was determined using the following formula: (% bead-positive SSC^high^, CD11R1^+^, and CD4^−^ cells × MFI of bead-positive cells)/10,000, where MFI is mean fluorescence intensity.

## References

[1] Stamatatos L, Morris L, Burton DR, Mascola JR 2009 Neutralizing antibodies generated during natural HIV-1 infection: good news for an HIV-1 vaccine? Nat Med 15:866–870. doi:10.1038/nm.1949. 19525964

[2] Doria-Rose NA, Klein RM, Manion MM, O'Dell S, Phogat A, Chakrabarti B, Hallahan CW, Migueles SA, Wrammert J, Ahmed R, Nason M, Wyatt RT, Mascola JR, Connors M 2009 Frequency and phenotype of human immunodeficiency virus envelope-specific B cells from patients with broadly cross-neutralizing antibodies. J Virol 83:188–199. doi:10.1128/JVI.01583-08. 18922865 PMC2612342

[3] Simek MD, Rida W, Priddy FH, Pung P, Carrow E, Laufer DS, Lehrman JK, Boaz M, Tarragona-Fiol T, Miiro G, Birungi J, Pozniak A, McPhee DA, Manigart O, Karita E, Inwoley A, Jaoko W, DeHovitz J, Bekker LG, Pitisuttithum P, Paris R, Walker LM, Poignard P, Wrin T, Fast PE, Burton DR, Koff WC 2009 Human immunodeficiency virus type 1 elite neutralizers: individuals with broad and potent neutralizing activity identified by using a high-throughput neutralization assay together with an analytical selection algorithm. J Virol 83:7337–7348. doi:10.1128/JVI.00110-09. 19439467 PMC2704778

[4] Hraber P, Korber BT, Lapedes AS, Bailer RT, Seaman MS, Gao H, Greene KM, McCutchan F, Williamson C, Kim JH, Tovanabutra S, Hahn BH, Swanstrom R, Thomson MM, Gao F, Harris L, Giorgi E, Hengartner N, Bhattacharya T, Mascola JR, Montefiori DC 2014 Impact of clade, geography, and age of the epidemic on HIV-1 neutralization by antibodies. J Virol 88:12623–12643. doi:10.1128/JVI.01705-14. 25142591 PMC4248897

[5] Richman DD, Wrin T, Little SJ, Petropoulos CJ 2003 Rapid evolution of the neutralizing antibody response to HIV type 1 infection. Proc Natl Acad Sci U S A 100:4144–4149. doi:10.1073/pnas.0630530100. 12644702 PMC153062

[6] Derdeyn CA, Moore PL, Morris L 2014 Development of broadly neutralizing antibodies from autologous neutralizing antibody responses in HIV infection. Curr Opin HIV AIDS 9:210–216. doi:10.1097/COH.0000000000000057. 24662931 PMC4068799

[7] Liao H-X, Lynch R, Zhou T, Gao F, Alam SM, Boyd SD, Fire AZ, Roskin KM, Schramm CA, Zhang Z, Zhu J, Shapiro L, NISC Comparative Sequencing Program, Becker J, Benjamin B, Blakesley R, Bouffard G, Brooks S, Coleman H, Dekhtyar M, Gregory M, Guan X, Gupta J, Han J, Hargrove A, Ho S-L, Johnson T, Legaspi R, Lovett S, Maduro Q, Masiello C, Maskeri B, McDowell J, Montemayor C, Mullikin J, Park M, Riebow N, Schandler K, Schmidt B, Sison C, Stantripop M, Thomas J, Thomas P, Vemulapalli M, Young A, Mullikin JC, Gnanakaran S, Hraber P, Wiehe K, Kelsoe G, Yang G, Xia S-M, Montefiori DC, Parks R, Lloyd KE, Scearce RM, Soderberg KA, Cohen M, Kamanga G, Louder MK, Tran LM, Chen Y, Cai F, Chen S, Moquin S, Du X, Joyce MG, Srivatsan S, Zhang B, Zheng A, Shaw GM, Hahn BH, Kepler TB, Korber BTM, Kwong PD, Mascola JR, Haynes BF 2013 Co-evolution of a broadly neutralizing HIV-1 antibody and founder virus. Nature 496:469–476. doi:10.1038/nature12053. 23552890 PMC3637846

[8] Wibmer CK, Bhiman JN, Gray ES, Tumba N, Abdool Karim SS, Williamson C, Morris L, Moore PL 2013 Viral escape from HIV-1 neutralizing antibodies drives increased plasma neutralization breadth through sequential recognition of multiple epitopes and immunotypes. PLoS Pathog 9:e1003738–16. doi:10.1371/journal.ppat.1003738. 24204277 PMC3814426

[9] Bhiman JN, Anthony C, Doria-Rose NA, Karimanzira O, Schramm CA, Khoza T, Kitchin D, Botha G, Gorman J, Garrett NJ, Abdool Karim SS, Shapiro L, Williamson C, Kwong PD, Mascola JR, Morris L, Moore PL 2015 Viral variants that initiate and drive maturation of V1V2-directed HIV-1 broadly neutralizing antibodies. Nat Med 21:1332–1336. doi:10.1038/nm.3963. 26457756 PMC4637988

[10] Moore PL, Gray ES, Wibmer CK, Bhiman JN, Nonyane M, Sheward DJ, Hermanus T, Bajimaya S, Tumba NL, Abrahams M-R, Lambson BE, Ranchobe N, Ping L, Ngandu N, Abdool Karim Q, Abdool Karim SS, Swanstrom RI, Seaman MS, Williamson C, Morris L 2012 Evolution of an HIV glycan-dependent broadly neutralizing antibody epitope through immune escape. Nat Med 18:1688–1692. doi:10.1038/nm.2985. 23086475 PMC3494733

[11] Gao F, Bonsignori M, Liao H-X, Kumar A, Xia S-M, Lu X, Cai F, Hwang K-K, Song H, Zhou T, Lynch RM, Alam SM, Moody MA, Ferrari G, Berrong M, Kelsoe G, Shaw GM, Hahn BH, Montefiori DC, Kamanga G, Cohen MS, Hraber P, Kwong PD, Korber BT, Mascola JR, Kepler TB, Haynes BF 2014 Cooperation of B cell lineages in induction of HIV-1-broadly neutralizing antibodies. Cell 158:481–491. doi:10.1016/j.cell.2014.06.022. 25065977 PMC4150607

[12] Doria-Rose NA, Schramm CA, Gorman J, Moore PL, Bhiman JN, DeKosky BJ, Ernandes MJ, Georgiev IS, Kim HJ, Pancera M, Staupe RP, Altae-Tran HR, Bailer RT, Crooks ET, Cupo A, Druz A, Garrett NJ, Hoi KH, Kong R, Louder MK, Longo NS, McKee K, Nonyane M, O'Dell S, Roark RS, Rudicell RS, Schmidt SD, Sheward DJ, Soto C, Wibmer CK, Yang Y, Zhang Z, NISC Comparative Sequencing Program, Mullikin JC, Binley JM, Sanders RW, Wilson IA, Moore JP, Ward AB, Georgiou G, Williamson C, Karim SSA, Morris L, Kwong PD, Shapiro L, Mascola JR 2014 Developmental pathway for potent V1V2-directed HIV-neutralizing antibodies. Nature 509:55–62. doi:10.1038/nature13036. 24590074 PMC4395007

[13] Wu X, Zhang Z, Schramm CA, Joyce MG, Do Kwon Y, Zhou T, Sheng Z, Zhang B, O'Dell S, McKee K, Georgiev IS, Chuang G-Y, Longo NS, Lynch RM, Saunders KO, Soto C, Srivatsan S, Yang Y, Bailer RT, Louder MK, NISC Comparative Sequencing Program, Benjamin B, Blakesley R, Bouffard G, Brooks S, Coleman H, Dekhtyar M, Gregory M, Guan X, Gupta J, Han J, Hargrove A, Ho S-L, Legaspi R, Maduro Q, Masiello C, Maskeri B, McDowell J, Montemayor C, Park M, Riebow N, Schandler K, Schmidt B, Sison C, Stantripop M, Thomas J, Thomas P, Vemulapalli M, Young A, Mullikin JC, Connors M, Kwong PD, Mascola JR, Shapiro L 2015 Maturation and diversity of the VRC01-antibody lineage over 15 years of chronic HIV-1 infection. Cell 161:470–485. doi:10.1016/j.cell.2015.03.004. 25865483 PMC4706178

[14] Buchbinder SP, Mehrotra DV, Duerr A, Fitzgerald DW, Mogg R, Li D, Gilbert PB, Lama JR, Marmor M, del Rio C, McElrath MJ, Casimiro DR, Gottesdiener KM, Chodakewitz JA, Corey L, Robertson MN 2008 Efficacy assessment of a cell-mediated immunity HIV-1 vaccine (the Step Study): a double-blind, randomised, placebo-controlled, test-of-concept trial. Lancet 372:1881–1893. doi:10.1016/S0140-6736(08)61591-3. 19012954 PMC2721012

[15] Gray GE, Allen M, Moodie Z, Churchyard G, Bekker L-G, Nchabeleng M, Mlisana K, Metch B, de Bruyn G, Latka MH, Roux S, Mathebula M, Naicker N, Ducar C, Carter DK, Puren A, Eaton N, McElrath MJ, Robertson M, Corey L, Kublin JG, HVTN 503/Phambili study team. 2011 Safety and efficacy of the HVTN 503/Phambili study of a clade-B-based HIV-1 vaccine in South Africa: a double-blind, randomised, placebo-controlled test-of-concept phase 2b study. Lancet Infect Dis 11:507–515. doi:10.1016/S1473-3099(11)70098-6. 21570355 PMC3417349

[16] Rerks-Ngarm S, Pitisuttithum P, Nitayaphan S, Kaewkungwal J, Chiu J, Paris R, Premsri N, Namwat C, de Souza M, Adams E, Benenson M, Gurunathan S, Tartaglia J, McNeil JG, Francis DP, Stablein D, Birx DL, Chunsuttiwat S, Khamboonruang C, Thongcharoen P, Robb ML, Michael NL, Kunasol P, Kim JH 2009 Vaccination with ALVAC and AIDSVAX to prevent HIV-1 infection in Thailand. N Engl J Med 361:2209–2220. doi:10.1056/NEJMoa0908492. 19843557

[17] Hammer SM, Sobieszczyk ME, Janes H, Karuna ST, Mulligan MJ, Grove D, Koblin BA, Buchbinder SP, Keefer MC, Tomaras GD, Frahm N, Hural J, Anude C, Graham BS, Enama ME, Adams E, DeJesus E, Novak RM, Frank I, Bentley C, Ramirez S, Fu R, Koup RA, Mascola JR, Nabel GJ, Montefiori DC, Kublin J, McElrath MJ, Corey L, Gilbert PB 2013 Efficacy trial of a DNA/rAd5 HIV-1 preventive vaccine. N Engl J Med 369:2083–2092. doi:10.1056/NEJMoa1310566. 24099601 PMC4030634

[18] Seaman MS, LeBlanc DF, Grandpre LE, Bartman MT, Montefiori DC, Letvin NL, Mascola JR 2007 Standardized assessment of NAb responses elicited in rhesus monkeys immunized with single- or multi-clade HIV-1 envelope immunogens. Virology 367:175–186. doi:10.1016/j.virol.2007.05.024. 17599382 PMC2075526

[19] Vaine M, Wang S, Hackett A, Arthos J, Lu S 2010 Antibody responses elicited through homologous or heterologous prime-boost DNA and protein vaccinations differ in functional activity and avidity. Vaccine 28:2999–3007. doi:10.1016/j.vaccine.2010.02.006. 20170767 PMC2847033

[20] Nkolola JP, Bricault CA, Cheung A, Shields J, Perry J, Kovacs JM, Giorgi E, van Winsen M, Apetri A, Brinkman-van der Linden ECM, Chen B, Korber B, Seaman MS, Barouch DH 2014 Characterization and immunogenicity of a novel mosaic M HIV-1 gp140 trimer. J Virol 88:9538–9552. doi:10.1128/JVI.01739-14. 24965452 PMC4136343

[21] Bricault CA, Kovacs JM, Nkolola JP, Yusim K, Giorgi EE, Shields JL, Perry J, Lavine CL, Cheung A, Ellingson-Strouss K, Rademeyer C, Gray GE, Williamson C, Stamatatos L, Seaman MS, Korber BT, Chen B, Barouch DH 2015 A multivalent clade C HIV-1 Env trimer cocktail elicits a higher magnitude of neutralizing antibodies than any individual component. J Virol 89:2507–2519. doi:10.1128/JVI.03331-14. 25540368 PMC4325749

[22] Malherbe DC, Doria-Rose NA, Misher L, Beckett T, Puryear WB, Schuman JT, Kraft Z, O'Malley J, Mori M, Srivastava I, Barnett S, Stamatatos L, Haigwood NL 2011 Sequential immunization with a subtype B HIV-1 envelope quasispecies partially mimics the in vivo development of neutralizing antibodies. J Virol 85:5262–5274. doi:10.1128/JVI.02419-10. 21430056 PMC3094990

[23] Bradley T, Fera D, Bhiman J, Eslamizar L, Lu X, Anasti K, Zhang R, Sutherland LL, Scearce RM, Bowman CM, Stolarchuk C, Lloyd KE, Parks R, Eaton A, Foulger A, Nie X, Karim SSA, Barnett S, Kelsoe G, Kepler TB, Alam SM, Montefiori DC, Moody MA, Liao H-X, Morris L, Santra S, Harrison SC, Haynes BF 2016 Structural constraints of vaccine-induced tier-2 autologous HIV neutralizing antibodies targeting the receptor-binding site. Cell Rep 14:43–54. doi:10.1016/j.celrep.2015.12.017. 26725118 PMC4706810

[24] McGuire AT, Hoot S, Dreyer AM, Lippy A, Stuart A, Cohen KW, Jardine J, Menis S, Scheid JF, West AP, Schief WR, Stamatatos L 2013 Engineering HIV envelope protein to activate germline B cell receptors of broadly neutralizing anti-CD4 binding site antibodies. J Exp Med 210:655–663. doi:10.1084/jem.20122824. 23530120 PMC3620356

[25] Jardine J, Julien JP, Menis S, Ota T, Kalyuzhniy O, McGuire A, Sok D, Huang PS, MacPherson S, Jones M, Nieusma T, Mathison J, Baker D, Ward AB, Burton DR, Stamatatos L, Nemazee D, Wilson IA, Schief WR 2013 Rational HIV immunogen design to target specific germline B cell receptors. Science 340:711–716. doi:10.1126/science.1234150. 23539181 PMC3689846

[26] Tian M, Cheng C, Chen X, Duan H, Cheng H-L, Dao M, Sheng Z, Kimble M, Wang L, Lin S, Schmidt SD, Du Z, Joyce MG, Chen Y, DeKosky BJ, Chen Y, Normandin E, Cantor E, Chen RE, Doria-Rose NA, Zhang Y, Shi W, Kong W-P, Choe M, Henry AR, Laboune F, Georgiev IS, Huang P-Y, Jain S, McGuire AT, Georgeson E, Menis S, Douek DC, Schief WR, Stamatatos L, Kwong PD, Shapiro L, Haynes BF, Mascola JR, Alt FW 2016 Induction of HIV neutralizing antibody lineages in mice with diverse precursor repertoires. Cell 166:1471–1484.e18. doi:10.1016/j.cell.2016.07.029. 27610571 PMC5103708

[27] Sanders RW, van Gils MJ, Derking R, Sok D, Ketas TJ, Burger JA, Ozorowski G, Cupo A, Simonich C, Goo L, Arendt H, Kim HJ, Lee JH, Pugach P, Williams M, Debnath G, Moldt B, van Breemen MJ, Isik G, Medina-Ramirez M, Back JW, Koff WC, Julien JP, Rakasz EG, Seaman MS, Guttman M, Lee KK, Klasse PJ, LaBranche C, Schief WR, Wilson IA, Overbaugh J, Burton DR, Ward AB, Montefiori DC, Dean H, Moore JP 2015 HIV-1 neutralizing antibodies induced by native-like envelope trimers. Science 349:aac4223. doi:10.1126/science.aac4223. 26089353 PMC4498988

[28] de Taeye SW, Ozorowski G, Torrents de la Peña A, Guttman M, Julien J-P, van den Kerkhof TLGM, Burger JA, Pritchard LK, Pugach P, Yasmeen A, Crampton J, Hu J, Bontjer I, Torres JL, Arendt H, DeStefano J, Koff WC, Schuitemaker H, Eggink D, Berkhout B, Dean H, LaBranche C, Crotty S, Crispin M, Montefiori DC, Klasse PJ, Lee KK, Moore JP, Wilson IA, Ward AB, Sanders RW 2015 Immunogenicity of stabilized HIV-1 envelope trimers with reduced exposure of non-neutralizing epitopes. Cell 163:1702–1715. doi:10.1016/j.cell.2015.11.056. 26687358 PMC4732737

[29] McCoy LE, van Gils MJ, Ozorowski G, Messmer T, Briney B, Voss JE, Kulp DW, Macauley MS, Sok D, Pauthner M, Menis S, Cottrell CA, Torres JL, Hsueh J, Schief WR, Wilson IA, Ward AB, Sanders RW, Burton DR 2016 Holes in the glycan shield of the native HIV envelope are a target of trimer-elicited neutralizing antibodies. Cell Rep 16:2327–2338. doi:10.1016/j.celrep.2016.07.074. 27545891 PMC5007210

[30] Pauthner M, Havenar-Daughton C, Sok D, Nkolola JP, Bastidas R, Boopathy AV, Carnathan DG, Chandrashekar A, Cirelli KM, Cottrell CA, Eroshkin AM, Guenaga J, Kaushik K, Kulp DW, Liu J, McCoy LE, Oom AL, Ozorowski G, Post KW, Sharma SK, Steichen JM, de Taeye SW, Tokatlian T, Torrents de la Peña A, Butera ST, LaBranche CC, Montefiori DC, Silvestri G, Wilson IA, Irvine DJ, Sanders RW, Schief WR, Ward AB, Wyatt RT, Barouch DH, Crotty S, Burton DR 2017 Elicitation of robust tier 2 neutralizing antibody responses in nonhuman primates by HIV envelope trimer immunization using optimized approaches. Immunity 46:1073–1088.e6. doi:10.1016/j.immuni.2017.05.007. 28636956 PMC5483234

[31] Saunders KO, Nicely NI, Wiehe K, Bonsignori M, Meyerhoff RR, Parks R, Walkowicz WE, Aussedat B, Wu NR, Cai F, Vohra Y, Park PK, Eaton A, Go EP, Sutherland LL, Scearce RM, Barouch DH, Zhang R, Holle Von T, Overman RG, Anasti K, Sanders RW, Moody MA, Kepler TB, Korber B, Desaire H, Santra S, Letvin NL, Nabel GJ, Montefiori DC, Tomaras GD, Liao H-X, Alam SM, Danishefsky SJ, Haynes BF 2017 Vaccine elicitation of high mannose-dependent neutralizing antibodies against the v3-glycan broadly neutralizing epitope in nonhuman primates. Cell Rep 18:2175–2188. doi:10.1016/j.celrep.2017.02.003. 28249163 PMC5408352

[32] Nkolola JP, Peng H, Settembre EC, Freeman M, Grandpre LE, Devoy C, Lynch DM, La Porte A, Simmons NL, Bradley R, Montefiori DC, Seaman MS, Chen B, Barouch DH 2010 Breadth of neutralizing antibodies elicited by stable, homogeneous clade A and clade C HIV-1 gp140 envelope trimers in guinea pigs. J Virol 84:3270–3279. doi:10.1128/JVI.02252-09. 20053749 PMC2838122

[33] Kovacs JM, Nkolola JP, Peng H, Cheung A, Perry J, Miller CA, Seaman MS, Barouch DH, Chen B 2012 HIV-1 envelope trimer elicits more potent neutralizing antibody responses than monomeric gp120. Proc Natl Acad Sci U S A 109:12111–12116. doi:10.1073/pnas.1204533109. 22773820 PMC3409750

[34] Fischer W, Perkins S, Theiler J, Bhattacharya T, Yusim K, Funkhouser R, Kuiken C, Haynes B, Letvin NL, Walker BD, Hahn BH, Korber BT 2007 Polyvalent vaccines for optimal coverage of potential T-cell epitopes in global HIV-1 variants. Nat Med 13:100–106. doi:10.1038/nm1461. 17187074

[35] Scheid JF, Mouquet H, Ueberheide B, Diskin R, Klein F, Oliveira TYK, Pietzsch J, Fenyo D, Abadir A, Velinzon K, Hurley A, Myung S, Boulad F, Poignard P, Burton DR, Pereyra F, Ho DD, Walker BD, Seaman MS, Bjorkman PJ, Chait BT, Nussenzweig MC 2011 Sequence and structural convergence of broad and potent HIV antibodies that mimic CD4 binding. Science 333:1633–1637. doi:10.1126/science.1207227. 21764753 PMC3351836

[36] McLellan JS, Pancera M, Carrico C, Gorman J, Julien J-P, Khayat R, Louder R, Pejchal R, Sastry M, Dai K, O'Dell S, Patel N, Shahzad-ul Hussan S, Yang Y, Zhang B, Zhou T, Zhu J, Boyington JC, Chuang G-Y, Diwanji D, Georgiev I, Do Kwon Y, Lee D, Louder MK, Moquin S, Schmidt SD, Yang Z-Y, Bonsignori M, Crump JA, Kapiga SH, Sam NE, Haynes BF, Burton DR, Koff WC, Walker LM, Phogat S, Wyatt R, Orwenyo J, Wang L-X, Arthos J, Bewley CA, Mascola JR, Nabel GJ, Schief WR, Ward AB, Wilson IA, Kwong PD 2011 Structure of HIV-1 gp120 V1/V2 domain with broadly neutralizing antibody PG9. Nature 480:336–343. doi:10.1038/nature10696. 22113616 PMC3406929

[37] Mouquet H, Scharf L, Euler Z, Liu Y, Eden C, Scheid JF, Halper-Stromberg A, Gnanapragasam PNP, Spencer DIR, Seaman MS, Schuitemaker H, Feizi T, Nussenzweig MC, Bjorkman PJ 2012 Complex-type N-glycan recognition by potent broadly neutralizing HIV antibodies. Proc Natl Acad Sci U S A 109:E3268–E3277. doi:10.1073/pnas.1217207109. 23115339 PMC3511153

[38] Gorny MK, Conley AJ, Karwowska S, Buchbinder A, Xu JY, Emini EA, Koenig S, Zolla-Pazner S 1992 Neutralization of diverse human immunodeficiency virus type 1 variants by an anti-V3 human monoclonal antibody. J Virol 66:7538–7542. 1433529 10.1128/jvi.66.12.7538-7542.1992 PMC240465

[39] Stephenson KE, Neubauer GH, Reimer U, Pawlowski N, Knaute T, Zerweck J, Korber BT, Barouch DH 2015 Quantification of the epitope diversity of HIV-1-specific binding antibodies by peptide microarrays for global HIV-1 vaccine development. J Immunol Methods 416:105–123. doi:10.1016/j.jim.2014.11.006. 25445329 PMC4324361

[40] Pinter A, Honnen WJ, Kayman SC, Trochev O, Wu Z 1998 Potent neutralization of primary HIV-1 isolates by antibodies directed against epitopes present in the V1/V2 domain of HIV-1 gp120. Vaccine 16:1803–1811. doi:10.1016/S0264-410X(98)00182-0. 9795384

[41] Kayman SC, Wu Z, Revesz K, Chen H, Kopelman R, Pinter A 1994 Presentation of native epitopes in the V1/V2 and V3 regions of human immunodeficiency virus type 1 gp120 by fusion glycoproteins containing isolated gp120 domains. J Virol 68:400–410. 7504740 10.1128/jvi.68.1.400-410.1994 PMC236300

[42] Sarzotti-Kelsoe M, Bailer RT, Turk E, Lin C-L, Bilska M, Greene KM, Gao H, Todd CA, Ozaki DA, Seaman MS, Mascola JR, Montefiori DC 2014 Optimization and validation of the TZM-bl assay for standardized assessments of neutralizing antibodies against HIV-1. J Immunol Methods 409:131–146. doi:10.1016/j.jim.2013.11.022. 24291345 PMC4040342

[43] Seaman MS, Janes H, Hawkins N, Grandpre LE, Devoy C, Giri A, Coffey RT, Harris L, Wood B, Daniels MG, Bhattacharya T, Lapedes A, Polonis VR, McCutchan FE, Gilbert PB, Self SG, Korber BT, Montefiori DC, Mascola JR 2010 Tiered categorization of a diverse panel of HIV-1 Env pseudoviruses for assessment of neutralizing antibodies. J Virol 84:1439–1452. doi:10.1128/JVI.02108-09. 19939925 PMC2812321

[44] deCamp A, Hraber P, Bailer RT, Seaman MS, Ochsenbauer C, Kappes J, Gottardo R, Edlefsen P, Self S, Tang H, Greene K, Gao H, Daniell X, Sarzotti-Kelsoe M, Gorny MK, Zolla-Pazner S, LaBranche CC, Mascola JR, Korber BT, Montefiori DC 2014 Global panel of HIV-1 Env reference strains for standardized assessments of vaccine-elicited neutralizing antibodies. J Virol 88:2489–2507. doi:10.1128/JVI.02853-13. 24352443 PMC3958090

[45] Gaschen B, Taylor J, Yusim K, Foley B, Gao F, Lang D, Novitsky V, Haynes B, Hahn BH, Bhattacharya T, Korber B 2002 Diversity considerations in HIV-1 vaccine selection. Science 296:2354–2360. doi:10.1126/science.1070441. 12089434

[46] Korber B, Gnanakaran S 2009 The implications of patterns in HIV diversity for neutralizing antibody induction and susceptibility. Curr Opin HIV AIDS 4:408–417. doi:10.1097/COH.0b013e32832f129e. 20048705 PMC6426297

[47] Javaherian K, Langlois AJ, McDanal C, Ross KL, Eckler LI, Jellis CL, Profy AT, Rusche JR, Bolognesi DP, Putney SD 1989 Principal neutralizing domain of the human immunodeficiency virus type 1 envelope protein. Proc Natl Acad Sci U S A 86:6768–6772. 2771954 10.1073/pnas.86.17.6768 PMC297927

[48] Kovacs JM, Noeldeke E, Ha HJ, Peng H, Rits-Volloch S, Harrison SC, Chen B 2014 Stable, uncleaved HIV-1 envelope glycoprotein gp140 forms a tightly folded trimer with a native-like structure. Proc Natl Acad Sci U S A 111:18542–18547. doi:10.1073/pnas.1422269112. 25512514 PMC4284565

[49] Liu Y, Pan J, Cai Y, Grigorieff N, Harrison SC, Chen B 2017 Conformational states of a soluble, uncleaved HIV-1 envelope trimer. J Virol 91:e00175-17. doi:10.1128/JVI.00175-17. 28250125 PMC5411591

[50] Jiang X, Burke V, Totrov M, Williams C, Cardozo T, Gorny MK, Zolla-Pazner S, Kong X-P 2010 Conserved structural elements in the V3 crown of HIV-1 gp120. Nat Struct Mol Biol 17:955–961. doi:10.1038/nsmb.1861. 20622876

[51] Wu L, Yang Z-Y, Xu L, Welcher B, Winfrey S, Shao Y, Mascola JR, Nabel GJ 2006 Cross-clade recognition and neutralization by the V3 region from clade C human immunodeficiency virus-1 envelope. Vaccine 24:4995–5002. doi:10.1016/j.vaccine.2006.03.083. 16690178

[52] Gorny MK, Williams C, Volsky B, Revesz K, Wang XH, Burda S, Kimura T, Konings FAJ, Nadas A, Anyangwe CA, Nyambi P, Krachmarov C, Pinter A, Zolla-Pazner S 2006 Cross-clade neutralizing activity of human anti-V3 monoclonal antibodies derived from the cells of individuals infected with non-B clades of human immunodeficiency virus type 1. J Virol 80:6865–6872. doi:10.1128/JVI.02202-05. 16809292 PMC1489067

[53] Zolla-Pazner S, Kong XP, Jiang X, Cardozo T, Nadas A, Cohen S, Totrov M, Seaman MS, Wang S, Lu S 2011 Cross-clade HIV-1 neutralizing antibodies induced with V3-scaffold protein immunogens following priming with gp120 DNA. J Virol 85:9887–9898. doi:10.1128/JVI.05086-11. 21795338 PMC3196418

[54] Sok D, Le KM, Vadnais M, Saye-Francisco KL, Jardine JG, Torres JL, Berndsen ZT, Kong L, Stanfield R, Ruiz J, Ramos A, Liang C-H, Chen PL, Criscitiello MF, Mwangi W, Wilson IA, Ward AB, Smider VV, Burton DR 2017 Rapid elicitation of broadly neutralizing antibodies to HIV by immunization in cows. Nature 548:108–111. doi:10.1038/nature23301. 28726771 PMC5812458

[55] Barouch DH, Alter G, Broge T, Linde C, Ackerman ME, Brown EP, Borducchi EN, Smith KM, Nkolola JP, Liu J, Shields J, Parenteau L, Whitney JB, Abbink P, Ng‘ang'a DM, Seaman MS, Lavine CL, Perry JR, Li W, Colantonio AD, Lewis MG, Chen B, Wenschuh H, Reimer U, Piatak M, Lifson JD, Handley SA, Virgin HW, Koutsoukos M, Lorin C, Voss G, Weijtens M, Pau MG, Schuitemaker H 2015 Protective efficacy of adenovirus/protein vaccines against SIV challenges in rhesus monkeys. Science 349:320–324. doi:10.1126/science.aab3886. 26138104 PMC4653134

[56] Sanders RW, Derking R, Cupo A, Julien J-P, Yasmeen A, de Val N, Kim HJ, Blattner C, la Peña de AT, Korzun J, Golabek M, de los Reyes K, Ketas TJ, van Gils MJ, King CR, Wilson IA, Ward AB, Klasse PJ, Moore JP 2013 A next-generation cleaved, soluble HIV-1 Env trimer, BG505 SOSIP.664 gp140, expresses multiple epitopes for broadly neutralizing but not non-neutralizing antibodies. PLoS Pathog 9:e1003618. doi:10.1371/journal.ppat.1003618. 24068931 PMC3777863

[57] Frey G, Peng H, Rits-Volloch S, Morelli M, Cheng Y, Chen B 2008 A fusion-intermediate state of HIV-1 gp41 targeted by broadly neutralizing antibodies. Proc Natl Acad Sci U S A 105:3739–3744. doi:10.1073/pnas.0800255105. 18322015 PMC2268799

[58] Badamchi-Zadeh A, McKay PF, Korber BT, Barinaga G, Walters AA, Nunes A, Gomes JP, Follmann F, Tregoning JS, Shattock RJ 2016 A multi-component prime-boost vaccination regimen with a consensus MOMP antigen enhances Chlamydia trachomatis clearance. Front Immunol 7:162. doi:10.3389/fimmu.2016.00162. 27199987 PMC4848310

[59] Renard BY, Löwer M, Kühne Y, Reimer U, Rothermel A, Türeci Ö Castle JC, Sahin U 2011 rapmad: robust analysis of peptide microarray data. BMC Bioinformatics 12:324. doi:10.1186/1471-2105-12-324. 21816082 PMC3174949

[60] Liao HX, Tsao CY, Alam SM, Muldoon M, Vandergrift N, Ma BJ, Lu X, Sutherland LL, Scearce RM, Bowman C, Parks R, Chen H, Blinn JH, Lapedes A, Watson S, Xia SM, Foulger A, Hahn BH, Shaw GM, Swanstrom R, Montefiori DC, Gao F, Haynes BF, Korber B 2013 Antigenicity and immunogenicity of transmitted/founder, consensus, and chronic envelope glycoproteins of human immunodeficiency virus type 1. J Virol 87:4185–4201. doi:10.1128/JVI.02297-12. 23365441 PMC3624376

[61] Montefiori DC 2005 Evaluating neutralizing antibodies against HIV, SIV, and SHIV in luciferase reporter gene assays. Curr Protoc Immunol Chapter 12:Unit 12.11. doi:10.1002/0471142735.im1211s64. 18432938

[62] Wu X, Yang Z-Y, Li Y, Hogerkorp C-M, Schief WR, Seaman MS, Zhou T, Schmidt SD, Wu L, Xu L, Longo NS, McKee K, O'Dell S, Louder MK, Wycuff DL, Feng Y, Nason M, Doria-Rose N, Connors M, Kwong PD, Roederer M, Wyatt RT, Nabel GJ, Mascola JR 2010 Rational design of envelope identifies broadly neutralizing human monoclonal antibodies to HIV-1. Science 329:856–861. doi:10.1126/science.1187659. 20616233 PMC2965066

[63] Takizawa M, Chiba J, Haga S, Asano T, Yamazaki T, Yamamoto N, Honda M 2006 Novel two-parameter flow cytometry (MIL4/SSC followed by MIL4/CT7) allows for identification of five fractions of guinea pig leukocytes in peripheral blood and lymphoid organs. J Immunol Methods 311:47–56. doi:10.1016/j.jim.2006.01.010. 16533513

